# Glucose-6-phosphate dehydrogenase regulation in the hepatopancreas of the anoxia-tolerant marine mollusc, *Littorina littorea*

**DOI:** 10.7717/peerj.21

**Published:** 2013-02-12

**Authors:** Judeh L. Lama, Ryan A.V. Bell, Kenneth B. Storey

**Affiliations:** 1 Institute of Biochemistry and Department of Biology, Carleton University, Ottawa, Ontario, Canada K1S 5B6; 2 Institute of Biochemistry and Departments of Biology and Chemistry, Carleton University, Ottawa, Ontario, Canada K1S 5B6; 3 Institute of Biochemistry and Departments and Chemistry, Carleton University, Ottawa, Ontario, Canada K1S 5B6

**Keywords:** Pentose phosphate pathway, Anoxia, Littorina littorea, Reversible protein phosphorylation

## Abstract

Glucose-6-phosphate dehydrogenase (G6PDH) gates flux through the pentose phosphate pathway and is key to cellular antioxidant defense due to its role in producing NADPH. Good antioxidant defenses are crucial for anoxia-tolerant organisms that experience wide variations in oxygen availability. The marine mollusc, *Littorina littorea*, is an intertidal snail that experiences daily bouts of anoxia/hypoxia with the tide cycle and shows multiple metabolic and enzymatic adaptations that support anaerobiosis. This study investigated the kinetic, physical and regulatory properties of G6PDH from hepatopancreas of *L. littorea* to determine if the enzyme is differentially regulated in response to anoxia, thereby providing altered pentose phosphate pathway functionality under oxygen stress conditions. Several kinetic properties of G6PDH differed significantly between aerobic and 24 h anoxic conditions; compared with the aerobic state, anoxic G6PDH (assayed at pH 8) showed a 38% decrease in *K*_*m*_ G6P and enhanced inhibition by urea, whereas in pH 6 assays *K*_*m*_ NADP and maximal activity changed significantly between the two states. The mechanism underlying anoxia-responsive changes in enzyme properties proved to be a change in the phosphorylation state of G6PDH. This was documented with immunoblotting using an anti-phosphoserine antibody, *in vitro* incubations that stimulated endogenous protein kinases versus protein phosphatases and significantly changed *K*_*m*_ G6P, and phosphorylation of the enzyme with ^32^P-ATP. All these data indicated that the aerobic and anoxic forms of G6PDH were the high and low phosphate forms, respectively, and that phosphorylation state was modulated in response to selected endogenous protein kinases (PKA or PKG) and protein phosphatases (PP1 or PP2C). Anoxia-induced changes in the phosphorylation state of G6PDH may facilitate sustained or increased production of NADPH to enhance antioxidant defense during long term anaerobiosis and/or during the transition back to aerobic conditions when the reintroduction of oxygen causes a rapid increase in oxidative stress.

## Introduction

Glucose-6-phosphate dehydrogenase (G6PDH; E.C. 1.1.1.49) catalyzes the rate determining step of the pentose phosphate pathway (PPP) and is the first of two oxidative steps within that pathway:

\begin{eqnarray*} \displaystyle {\text{D-glucose-6-phosphate + NADP}}^{+}&&\displaystyle \end{eqnarray*}

\begin{eqnarray*} \displaystyle \rightarrow \text{D-6-phosphoglucono-}\delta {\text{-lactone + NADPH + H}}^{+}.&&\displaystyle \end{eqnarray*}

The pentose phosphate pathway has several important functions in cells including providing sugars for the synthesis of aromatic amino acids and nucleotides and producing NADPH reducing equivalents that are used in biosynthesis and antioxidant defense. With respect to antioxidant defense the NADPH generated by the pentose phosphate pathway is the main source of cellular reducing equivalents that are used to regenerate the reduced forms of glutathione and thioredoxin after they have been oxidized in antioxidant reactions (Riganti et al., 2012).

Protection against oxidative damage is important in organisms that experience wide fluctuations in environmental oxygen content, particularly to deal with transitions from low to high oxygen because a rapid increase in oxygen availability and/or consumption is paralleled by a comparable increase in reactive oxygen species (ROS) generation (Hermes-Lima, Storey & Storey, 1998). In addition, many organisms that naturally endure prolonged episodes of hypoxia/anoxia do so because they use a strategy of metabolic rate depression. By regulating a strong suppression of metabolism (often to less than 10% of normoxic rate), they gain a comparable extension (e.g. 10-fold) of the time that they can survive using endogenous fuels and fermentative pathways (glycolysis) to produce energy (Brooks & Storey, 1997; Larade & Storey, 2009). However, when oxygen becomes available again, oxgen availability, oxygen consumption and ROS production all rise rapidly.

Cycles of high versus low oxygen availablity are a characteristic of life in the marine intertidal zone. For example, for gill-breathing snails such as the common periwinkle, *Littorina littorea*, oxygen is plentiful when snails are immersed in water but falls when snails are out of water at low tide. Oxygen can also vary widely for animals trapped in tide pools at low tide and in winter the snails living on northern seashores also undergo freeze/thaw cycles and while frozen there is no delivery of oxygen to tissues (Larade & Storey, 2009). Furthermore, environmental conditions, such as predation, high salinity, or high levels of toxins can force snails to close their shell opening enforcing extended periods of hypoxia/anoixa (Truchot & Duhamel-Jouve, 1980; de Zwaan & Putzer, 1985).

With this background, we proposed that G6PDH, with its key role in NADPH generation for antioxidant defense, may be differentially regulated between aerobic and anoxic states to adjust for changing circumstances that may include (a) a net suppression of pentose phosphate flux as a part of overall metabolic rate depression, (b) a probable shift in the types of pathways requiring NADPH to de-emphasize ATP-expensive biosynthesis (e.g. lipogenesis) as compared with other uses for NADPH such as antioxidant defense. The present study examines the physical and kinetic characteristics of G6PDH from hepatopancreas of *L. littorea* exposed to aerobic versus anoxic conditions, and evaluates reversible protein phosphorylation as a regulatory mechanism that may provide differential control of G6PDH in the two states.

## Materials and Methods

### Animals

Common periwinkles (*Littorina littorea*) were purchased from the Kowloon Market, Ottawa. The snails were placed in a 30 L tub of aerated, full strength seawater (1000 mOsmol/L made using Instant Ocean Sea Salt; salinity confirmed with a buoyancy meter) at 9 °C in an incubator with constant darkness. No food was provided but the water was changed periodically and any dead snails were removed. Experiments to gather aerobic control (sampled directly from the seawater) and 24 h anoxia-exposed snails were commenced after 7 days of acclimation.

For anoxia exposure, 25 snails were placed in each of several sealed jars that were fitted with two gas ports. The containers were held on ice and contained a small amount of deoxygenated seawater (1 cm deep) which had been bubbled previously with N_2_ gas for 30 min before addition to the jars. The lids were tightened and sealed with parafilm and then a gas line was connected to one of the ports whereas the other port was opened to vent the gas. N_2_ gas was continuously bubbled into the water in the jars for a further 20 min. Then the N_2_ line was removed, the ports were closed and the containers were returned to the 9 °C incubator for a 24 h anoxia exposure.

For sampling of anoxic snails, a container of snails was removed from the incubator, placed on ice and nitrogen gassing was reinstated. Snails were sampled from the jar, quickly dissected, and hepatopancreas samples were immediately frozen in liquid nitrogen. Tissues were stored at −80 °C until use.

### Sample preparation

Frozen samples of hepatopancreas were homogenized 1:5 w:v in ice-cold homogenization buffer containing 50 mM Tris-HCl, pH 7.5, 10% v:v glycerol, 10 mM 2-mercaptoethanol, 25 mM NaF, 2.5 mM EDTA and 2.5 mM EGTA. The latter three buffer components provide inhibition of protein phosphatases and protein kinases to ensure that the phosphorylaion state of the enzyme does not change prior to assay. A few crystals of phenylmethylsulfonyl fluoride (PMSF), a protease inhibitor, were also added at the time of homogenization. Homogenates were centrifuged at 13,500 g for 30 min at 4 °C and the supernatant was decanted and held on ice.

### Sephadex G-50 filtration of crude extracts

To properly assess G6PDH properties, low molecular weight metabolites and ions were removed from the enzyme preparation by Sephadex G-50 gel filtration. A 5 cm column of Sephadex G-50 in a syringe barrel was equilibrated in homogenization buffer and centrifuged at 500 g in a bench-top centrifuge for 2 min to remove excess buffer which was discarded. Then a 500 µL aliquot of supernatant was layered on top, the column was centrifuged again and the protein-containing eluant was removed and stored on ice.

### G6PDH assays

G6PDH activity was measured spectrophotometrically by monitoring the production of NADPH (extinction coefficient of 6220 M^−1^ cm^−1^) at 340 nm using a Thermo Labsystems Multiskan Spectrum Microplate Spectrophotometer. Optimal assay conditions were 4 mM G6P, 1.0 mM NADP^+^ , 50 mM Tris-HCl buffer, pH 8.0. Reactions were initiated by adding 5 µl of enzyme extract (10 µlfor incubation studies or 50 µl for assaying column fractions) to a 200 µL total reaction volume. Microplate Analysis (MPA) and Kinetics 3.51 computer programs were used to analyze data and obtain kinetic parameters (Brooks, 1994). One unit of G6PDH activity is defined as the amount of enzyme that produces 1 µmol 6-phosphogluconolactone per min at 22 °C and activities were expressed as mU/mg soluble protein. Soluble protein content was quantified using the Coomassie blue dye binding method and the Bio-Rad prepared reagent with a standard curve for bovine serum albumin.

### pH studies

A pH profile of G6PDH activity was conducted by measuring activity under optimal conditions at 22 °C for pH values ranging from 6.0 to 10.5. A 50 mM Bis-Tris Propane buffer was used for pH 6.0–9.0 and CAPS buffer was used for pH 9.5–10.5. Standard kinetic parameters were determined at pH 6.0 and pH 8.0 for hepatopancreas preparations from both aerobic control and 24 h anoxic conditions. For pH 6.0 assays, MES buffer was used for both homogenization and assay (with the same components as in the standard Tris homogenization and assay buffers).

### Urea denaturation

Hepatopancreas homogenates were prepared as previously described and G6PDH susceptibility to urea denaturation was assessed by incubating samples of crude hepatopancreas extract from control and anoxic snails with different concentrations of urea (0–1 M) in homogenization buffer, pH 8.0 for 24 h at 22 °C. After incubation, aliquots of sample were used to measure remaining G6PDH activity under standard assay conditions.

### Effect of ions and other metabolites on G6PDH activity

Sephadex G-50 filtered soluble protein extracts of hepatopancreas from aerobic and anoxic snails were used to assess the effects of NaCl, KCl, and NH_4_Cl on G6PDH activity at concentrations up to 2 M. Comparable effects of selected metabolites were assessed in both pH 6 and pH 8 assays; metabolites tested included L-alanine, L-aspartic acid, phosphoenolpyruvate (PEP), AMP and succinate (0–10 mM concentration range for each metabolite). Assays were performed at suboptimal susbtrate concentrations (0.15 mM NADP^+^ , 0.1 mM G6P, at pH 8).

### Temperature studies

Maximum G6PDH velocity was measured at ∼5 °C intervals between 5 °C and 30 °C. Arrhenius plots were constructed and the energy of activation (*E*_*A*_) was calculated. To alter reaction temperature, the microplate reader was placed in a Precision Low Temperature Incubator 815 and temperature was adjusted to the desired values. Filled microplates were placed into the spectrophotometer and allowed to equilibrate to the desired temperature for several minutes before the assay was commenced by addition of enzyme. A telethermometer was used to measure microplate well temperature before and after assay.

### DEAE-sephadex elution profiles

DEAE-Sephadex columns (5 cm × 1 cm) were equilibrated in column buffer (25 mM Tris-HCl, 5% v:v glycerol, 5 mM 2-mercaptoethanol, 12.5 mM NaF, 1.25 mM EDTA, 1.25 mM EGTA, pH 7.0). A 300 µl aliquot of supernatant was added to each column, followed by washing with 10 mL of the same buffer to remove unbound proteins. The column was eluted with a linear gradient of 0–0.5 M KCl in 20 mL of column buffer. Hepatopancreas extracts from aerobic control and 24 h anoxia-exposed snails were run separately and fractions were collected with a Gilson FC203B fraction collector. G6PDH activity was assayed under optimal assay conditions using 50 µl from each fraction.

### Western blot analysis of G6PDH phosphorylation state

G6PDH was partially purified using affinity chromatography. A 1 mL aliquot of crude hepatopancreas extract was added to a blue-agarose column (3 cm × 1 cm,h × d) equilibrated in column buffer. G6PDH was then eluted with a 0–1 M KCl gradient. The fraction with the highest activity was selected and mixed 1:1 (v:v) with SDS loading buffer (100 mM Tris buffer, pH 6.8, 4% w:v SDS, 20% v:v glycerol, 0.2% w:v bromophenol blue and 10% v:v 2-mercaptoethanol) , boiled for 5 min, cooled on ice and frozen at −20 °C until use.

SDS resolving gels (8% v/v acrylamide, 400 mM Tris, pH 8.8, 0.1% w/v SDS, 0.2% w/v ammonium persulfate [APS], 0.04% v/v TEMED) were prepared with a 5% stacking gel (5% acrylamide, 190 mM Tris, pH 6.8, 0.1% w/v SDS, 0.15% w/v APS, 0.1% v/v TEMED). Partially purified G6PDH samples were loaded onto these gels and separated electrophoretically in SDS-PAGE running buffer (25 mM Tris-base, 190 mM glycine, and 0.1% w/v SDS) at 180 V for 45 min. A 3 µL aliquot of Spectra™ Multicolor Broad Range Protein Ladder was added to one lane of every gel. Following electrophoresis, proteins were electroblotted onto polyvinylidienedifluoride (PVDF) membranes (Millipore) by wet transfer. Electroblotting was perfomed at room temperature for 1.5 h at 160 mA in transfer buffer (25 mM Tris, pH 8.5, 192 mM glycine, and 20% v/v methanol).

Following protein transfer, PVDF membranes were incubated overnight at 4 °C with a phospho-serine primary antibody (Calbiochem) diluted at 1:1000 in Tris-buffered saline with Tween-20 (TBST; 20 mM Tris-base, 140 mM NaCl, 0.05% Tween-20) with a small amount of sodium azide added. Subsequently, membranes were washed with TBST three times for 5 min each, followed by incubation with anti-rabbit secondary antibody conjugated with horseradish peroxidase (Bioshop Canada) at a dilution of 1:4000 in TBST. Membranes were incubated at room temperature for 30 min and then washed three times for 5 min each with ddH_2_O. Bands were then detected using enzymatic chemiluminescence (ECL), initiated by the addition of 600 µL of hydrogen peroxide and 600 µL of luminol reagent to the membrane’s surface for several seconds followed by visualization using a ChemiGenius Bioimaging System (Syngene, MD, USA). Membranes were then stained with Coomassie blue (0.25% w/v Coomassie Brilliant Blue R in 50% methanol, 7.5% acetic acid) and destained with destaining solution (60% v/v methanol, 20% v/v acetic acid in ddH_2_O) until bands were clearly seen. Band densities were analyzed using Bioimaging software, and were standardized against the corresponding Coomassie blue stained bands. The G6PDH band on the phospho-serine Western blot was determined through a seperate Western blot which used an anti-G6PDH antibody (StressGen).

### In vitro incubations to stimulate endogenous protein kinases and phosphatases

The effects of endogenous protein kinases and phosphatases on G6PDH were analyzed by incubating hepatopancreas extracts from control and anoxic snails under conditions that stimulated the activities of specific endogenous protein kinases or protein phosphatases. Tissue extracts were prepared in standard homogenization buffer (denoted STOP). After centrifugation, 150 µl aliquots of soluble protein extract were incubated in 300 µl of the specific incubation solutions for 4 h at 4 °C. After incubation, samples were subjected to Sephadex G-50 gel filtration to remove metabolites that could interfere with the enzyme assay. *K*_*m*_ G6P values were determined under standard conditions at pH 8.0 useing 10 µl of incubation mixture for assay. The specific incubation solutions used were as follows:

STOP: standard homogenization buffer

Base Buffer: 50 mM Tris-HCl, pH 7.5, 10% glycerol, 10 mM 2-mercaptoethanol

To stimulate protein kinases:

Protein kinase A (PKA): base buffer  + 1 mM cAMP +5 mM Mg^.^ ATP +10 mM MgCl_2_ +30 mM NaF +5 mM Na_3_V O_4_

Protein kinase G (PKG): base buffer +1 mM cGMP +5 mM Mg^.^ ATP +10 mM MgCl_2_ +30 mM NaF +5 mM Na_3_V O_4_

Protein kinase C (PKC): base buffer +1.3 mM CaCl_2_ +7 ug/mL PMA (phorbol 12-myristate 13-acetate) +5 mM Mg^.^ATP +5 mM MgCl_2_ +30 mM NaF +5 mM Na_3_V O_4_

Total Kinase: base buffer +1 mM cAMP +5 mM Mg^.^ATP +1 mM cGMP +1.3 mM CaCl_2_ +7 ug/mL PMA (phorbol 12-myristate 13-acetate) +1 mM AMP +1 U of calmodulin (CaM) activity/incubation tube +10 mM MgCl_2_ +30 mM NaF +5 mM Na_3_V O_4_

To stimulate protein phosphatases

Protein phosphatase 1 (PP1): base buffer +no ions (therefore no PP2B or PP2C activity) +2.5 nM okadaic acid (inhibits PP2A) +5 mM Na_3_V O_4_ , 2 mM EDTA and 2 mM EGTA.

Protein phosphatases 1 +2A (PP1 +PP2A): base buffer +no ions (halts PP2B/PP2C activity) +30 mM Na_3_V O_4_ +2 mM EDTA +2 mM EGTA.

Protein phosphatase 2B (PP2B): base buffer +5 mM CaCl_2_ +2 mM EDTA (chelates Mg^2+^ , inhibits PP2C) +1 µM okadaic acid (inhibits PP1/PP2A) +5 mM Na_3_V O_4_

Protein phosphatase 2C (PP2C): base buffer +5 mM MgCl_2_ +2 mM EGTA (chelates Ca^2+^ , inhibits PP2B) +1 µM okadaic acid (inhibits PP1/PP2A) +5 mM Na_3_V O_4_ +1 nM cypermethrin (inhibits PP2B).

Total Phosphatase: base buffer +5 mM MgCl_2_ +5 mM CaCl_2_

### Immunoprecipitation of radiolabelled phosphorylated G6PDH

To confirm that incubations that stimulated protein kinases actually transfered phosphate groups onto G6PDH, these incubations were repeated in the presence of ^32^P-ATP. Then the radiolabelled phospho-G6PDH was immunoprecipitated using an anti-G6PDH antibody.

For optimal detection of phosphorylation, studies began with the dephosphorylated enzyme. Tissue extracts were prepared and low molecular weight solutes were removed via Sephadex G-50 filtration. Extracts were then incubated for 4 h under conditions that promoted Total Phosphatase action, as described above. Aliquots of dephosphorylated enzyme were then incubated under either STOP or Total Kinase incubation conditions (as above) with the inclusion of 5 µCi γ-^32^P-ATP in a 300 µl incubation volume, in addition to the regular amount of nonradioactive ATP. Following a 24 h incubation at 5 °C, 60 µl of insoluble Protein A (IPA; Sigma) suspension was added and further incubated overnight at 5 °C. Following this, samples were then centrifuged at 800 g in a Biofuge for 10 min to remove proteins that bound nonspecifically to Protein A and the supernatants were removed. Supernatant samples were then incubated with 1 µg G6PDH primary antibody (Sigma) for 1 h at 5 °C and then 60 µl of IPA was added and incubated overnight, again at 5 °C. Along with the G6PDH antibody, 50% polyethylene glycol-8000 was also added to the incubation tube to give a final PEG concentration of 5%. Following incubation, samples were centrifuged at 800 g for 10 min. Supernatants were decanted and the pellets were resuspended in 100 µl of 50 mM Tris buffer, pH 7.5. Pellet washing was repeated 7 times and after the final wash, 5 µl of each sample was spotted on P81 paper and exposed to a phosphorscreen for 1 h. Quantification of radioactivity was done using a Personal Molecular Imager^®^-FX (BioRad).

## Results

### G6PDH kinetic characterization

**Figure 1:**
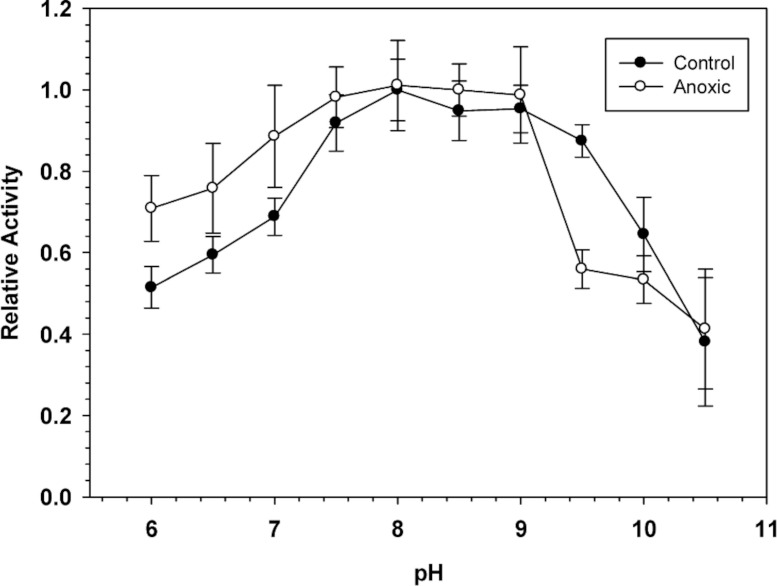
Effect of pH on the activity of hepatopancreas G6PDH from control and anoxic *L. littorea.* A 50 mM Bis-Tris Propane buffer was used for pH 6.0–9.0 and CAPS buffer was used for pH 9.5–10.5. Assays were run at 23 °C with the optimal substrate concentrations determined at pH 8. Data are relative values compared with activity at pH 8, means ± SEM, *n* = 4 independent determinations on separate tissue preparations.

To determine the optimal conditions for G6PDH activity, the enzyme was assayed over a wide range of pH. Figure 1 shows pH curves for hepatopancreas G6PDH from aerobic control and anoxic snails. Maximal activity occurred between pH 7.5 and 9 and therefore, standard assays were performed at pH 8.0 throughout the study, unless otherwise indicated.

Table 1 shows kinetic parameters for hepatopancreas G6PDH from aerobic control and anoxia-exposed snails and highlights several differences between the two enzyme forms. G6PDH showed Michaelis-Menten kinetics with hyperbolic velocity versus substrate concentration relationships for both G6P and NADPH. At pH 8.0 the Michaelis-Menten constant (*K*_*m*_) for G6P was significantly lower for the anoxic enzyme form, 38% lower than the corresponding value for the aerboic control enzyme. However, the *K*_*m*_ NADP and enzyme maximal velocity (*V*_max_) did not differ between the two enzyme forms. When assayed at pH 6.0, however, *K*_*m*_ G6P did not differ between aerobic and anoxic states but *K*_*m*_ NADP of anoxic G6PDH was 38% lower than the aerobic value whereas Vmax was 28% higher. An analysis of the effects of pH change on these parameters also revealed significant changes (Table 1). Analysis at the suboptimal pH of 6.0 resulted in strong significant increases in the *K*_*m*_ G6P of 4.2 and 7.8 fold and decreases in *V*_max_ to 66 and 84% for control and anoxic G6PDH, respectively, as well as a 1.6 fold increase in *K*_*m*_ NADP of control G6PDH.

**Table 1:** Kinetic parameters for hepatopancreas G6PDH from control and 24 h anoxic *L. littorea* analyzed at pH 8.0 and pH 6.0. Assays were performed at 22 °C and data are means ± SEM, *n* = 4 preparations from different animals. Kinetic constants were determined at optimal concentrations of ions or cosubstrates.

	Control	24 h Anoxic
Analysis at pH 8.0		
*K*_*m*_ G6P (mM)	0.097 ± 0.006	0.060 ± 0.003^a^
*K*_*m*_NADP^+^ (mM)	0.117 ± 0.006	0.115 ± 0.008
*V*_max_ (mU/mg)	30.0 ± 1.0	29.7 ± 0.8
Activation Energy, *E*_*A*_ (KJ/mol)	27 ± 3	22 ± 3
*I*_50_ Urea (mM)	90 ± 10	56 ± 3^a^
*I*_50_ NaCl (M)	0.99 ± 0.05	1.20 ± 0.10
*I*_50_ KCl (M)	1.32 ± 0.09	1.20 ± 0.20
*I*_50_NH_4_Cl (M)	0.9 ± 0.1	1.0 ± 0.1
Analysis at pH 6.0		
Km G6P (mM)	0.41 ± 0.04^b^	0.47 ± 0.02^b^
Km NADP (mM)	0.19 ± 0.02^b^	0.118 ± 0.008^a^
Vmax (mU/mg)	19.8 ± 0.6^b^	24.9 ± 1.3^a^ ^,^ ^b^

**Notes.**

a Significantly different from the control value using the Student’s t-test, *p* < 0.05.

b Significantly different from the corresponding value at pH 8.0, *p* < 0.025.

### Effects of urea, salts and metabolites on G6PDH activity

To assess potential urea denaturation of G6PDH, extracts of control and anoxic hepatopancreas were incubated for 24 h at 22 °C at multiple concentrations of urea ranging from 0–1 M. Subsequent assay of G6PDH activity allowed calculation of *I*_50_ values (inhibitor concentration that reduces activity by 50%). Table 1 shows that the anoxic form of G6PDH was more susceptible to urea denaturation than the aerobic control enzyme, with an *I*_50_ that was 38% lower than the control.

The effects of increasing NaCl, KCl or NH_4_Cl concentrations on G6PDH activity were also investigated. *I*_50_ values for all three salts were high (0.9–1.3 M) and no significant differences were seen between control and anoxic states (Table 1). A survey of the effects of selected metabolites (chosen because of their involvement in anaerobic metabolism in *L. littorea*) on hepatopancreas G6PDH from control and 24 h anoxic snails was also performed at pH 6 and 8. However, none of the metabolites tested (AMP, L-alanine, L-aspartic acid, succinate, phosphoenolpyruvate) had either positive or negative effects on enzyme activity (data not shown).

### Temperature studies

Activation energies for G6PDH were determined from Arrhenius plots for enzyme activities measured between 5 and 30 °C. The Arrhenius relationship was linear over the range of temperatures tested for the enzyme from both aerobic and anoxic states. The calculated activation energy, *E*_*A*_, for G6PDH from control hepatopancreas was 27 ± 3 kJ/mol which was not significantly different from the value for the enzyme from anoxic tissue, 22 ± 3 kJ/mol (Table 1).

### Ion exchange chromatography of G6PDH

**Figure 2:**
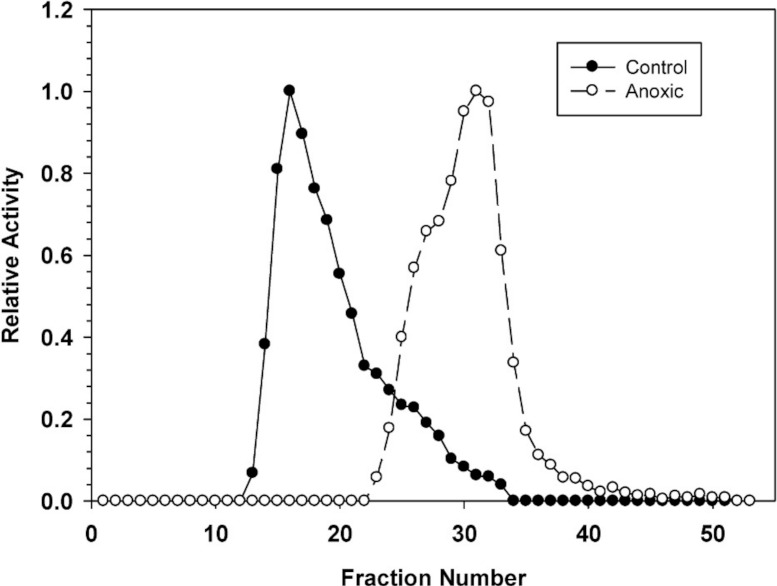
DEAE-Sephadex elution profiles for *L. littorea* hepatopancreas G6PDH activity from control and 24 h anoxic snails. Profiles are representative of three independent determinations for each condition. Activities are expressed relative to the highest activity fraction. G6PDH was eluted with a 0–0.5 M KCl gradient in buffer at pH 7.0.

Hepatopancreas extracts were subjected to DEAE-Sephadex ion exchange chromatography (Fig. 2). Development of the column with a 0–0.5 M salt gradient resulted in differential elution of G6PDH from aerobic and anoxic snails. The aerobic enzyme form eluted at a lower salt concentration (peak at fraction 16) whereas the anoxic enzyme showed peak elution at fraction 30. Aerobic extracts showed a substantial “tail” of activity from fractions 22–33 which may suggest that these fractions may contain a small percentage of the anoxic enzyme form.

### G6PDH phosphorylation state

**Figure 3:**
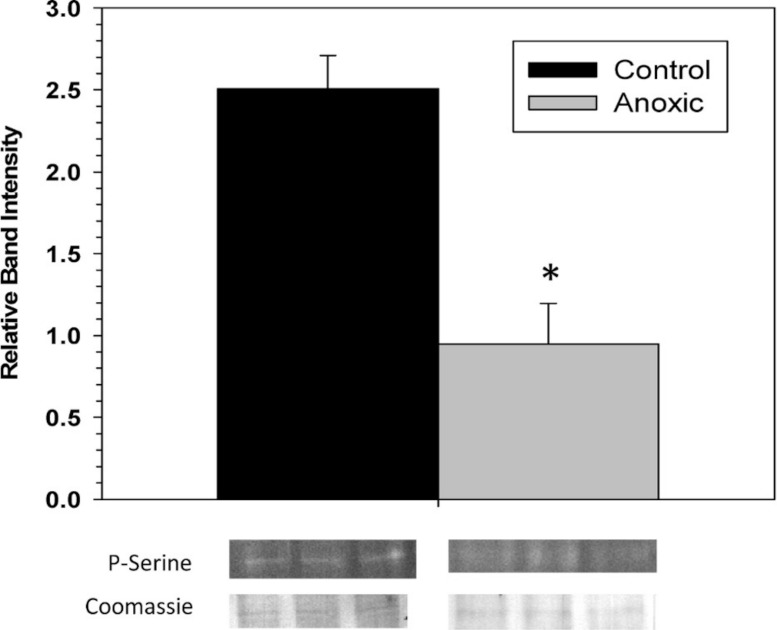
The serine phosphorylation state of hepatopancreas G6PDH from control and 24 h anoxic *L. littorea* as determined by Western blotting. Data are means ± SEM, *n* = 4 independent determinations. Bands beneath the bars are representative of typical of bands seen on Western blots. *- Significantly different from the control via the Student’s t-test, *p* < 0.05.

To examine the phosphorylation state of G6PDH the level of phosphorylated serine residues on G6PDH was determined via Western blotting on partially purified samples (the peak fraction from the blue-agarose column). This analysis found that phosphoserine band intensity was about 2.5 fold greater for aerobic G6PDH compared with the anoxic form (Fig. 3). A separate Western blot was conducted using an anti-G6PDH antibody (StressGen) to dictate the location of the G6PDH subunits.

**Figure 4:**
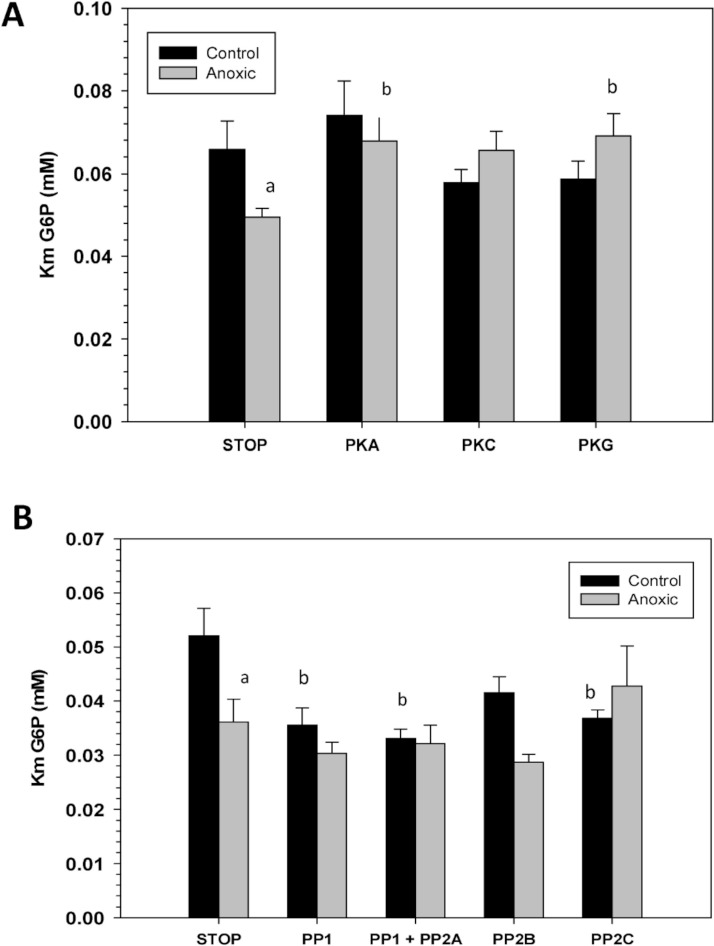
Effects of *in vitro* incubations to stimulate the activities of (A) protein kinases or (B) protein phosphatases on the *K*_*m*_ G6P of G6PDH from *L. littorea* hepatopancreas. Crude extracts were incubated for 4 h at 4 °C with different combinations of additives (as defined in the Materials and Methods) before assay at room temperature. Data are means + SEM, *n* = 3. a - Significantly different from the corresponding control value using the Student’s t-test, *P* < 0.05. b - Significantly different from the corresponding STOP value using ANOVA followed by the post-hoc Dunnett’s test, *P* < 0.05.

To assess the consequence of phosphorylation on G6PDH kinetics, aliquots of crude extracts of hepatopancreas from control and anoxic snails were incubated for 4 h at 4 °C under conditions that stimulated the activities of endogenous protein kinases or protein phosphatases and the effects on *K*_*m*_ G6P were determined. Figure 4A shows the effects of incubations that promote the actions of specific protein kinase on the *K*_*m*_ G6P. When incubated under conditions which stimulated PKA or PKG the anoxic form of G6PDH showed significant increases in *K*_*m*_ G6P, as compared to the corresponding *K*_*m*_ G6P when protein kinases were inhibited (STOP condition). However, treatments that stimulated kinase activities did not have any effect on the *K*_*m*_ G6P for G6PDH from aerobic control snails.

Conversely, when the effect of specific protein phosphatases on *K*_*m*_ G6P was investigated, *K*_*m*_ G6P for G6PDH in preparations from anoxic snails was not affected by any of the incubation treatments that stimulated endogenous protein phosphatase activities (Fig. 4B). However, stimulation of the activities of PP1, PP1 + PP2A, or PP2C all resulted in significant decreases in the *K*_*m*_ G6P of the aerobic control enzyme. *K*_*m*_ values decreased by approximately 30%, 40% and 30%, respectively, reducing these to values comparable to those of the anoxic enzyme under STOP conditions (Fig. 4B). Overall, these data from kinase and phosphatase incubations are consistent with the aerobic and anoxic G6PDH being the high and low phosphate forms of the enzyme, respectively.

**Figure 5:**
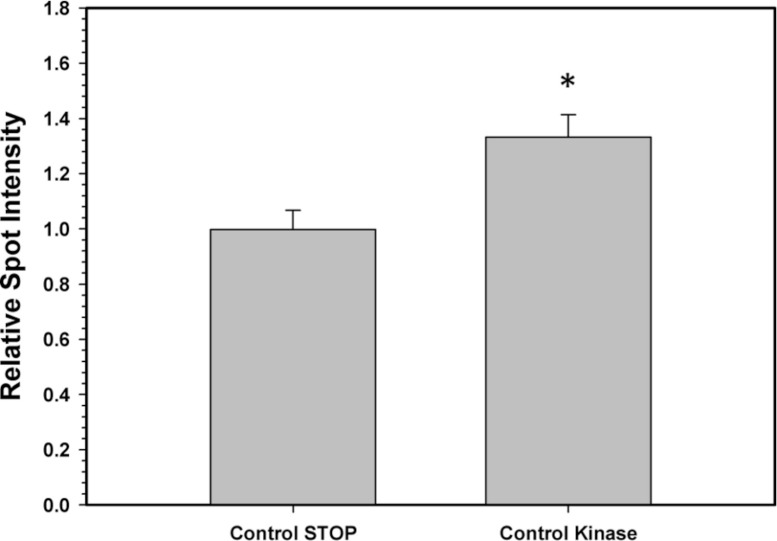
Radioactive immunoprecipitation of hepatopancreas G6PDH following *in vitro* incubations of the control enzyme to stimulate the activities of protein kinases. Data are means ± SEM, *n* = 3. * - significantly different from the corresponding STOP value using the Student’s t-test, *P* < 0.05.

To confirm that the above kinase incubations transfered phosphate groups onto G6PDH, the kinase incubations were repeated in the presence of γ-^32^P-ATP. The radiolabelled phospho-G6PDH was subsequently immunoprecipitated using insoluble protein A and a G6PDH antibody. Hepatopancreas extracts from aerobic control snails were first dephosphorylated by treatment under the Total Phosphatase conditions described in the Materials and Methods. Subsequently, the extracts were incubated with γ-^32^P-ATP under two conditions: STOP conditions that inhibit both kinases and phosphatases and Total Kinase conditions that promote the activity of several protein kinases (PKA, PKG, PKC, AMP-activated kinase, and Ca-calmodulin kinase). Figure 5 shows an approximately 30% increase in radioactivity incorporated onto G6PDH under Total Kinase conditions as compared with the respective STOP condition.

## Discussion

Intertidal molluscs are excellent facultative anaerobes and display multiple metabolic adaptations for anoxia survival including strong metabolic rate depression, large stores of fermentable fuels, and altered routes of anaerobic metabolism that generate more ATP than is possible from glycolysis alone (Brooks & Storey, 1997). They also display well developed antioxidant defenses that are both constitutive and stress-inducible (Freire et al., 2011). For example, antioxidant metabolites, such as pools of reduced glutathione, were elevated by 2–3 fold under anoxic conditions in *L. littorea* tissues to help protect cells (Pannunzio & Storey, 1998). Anoxia-induced enhancement of antioxidant defenses appears to result from the need to cope with a rapid, large increase in ROS formation associated with arousal from hypometabolism and the return to oxygen-based metabolism (Hermes-Lima, Storey & Storey, 1998) but a second reason may also be involved. More recent work suggests that well-developed antioxidant defenses are also important for long term protection of macromolecules during anaerobsis because the potential for degradation and resynthesis of oxidatively-damaged proteins is much reduced in the hypometabolic state (Storey & Storey, 2012).

The present study demonstrates that several enzymatic properties of hepatopancreas G6PDH differed significantly between control and anoxic states. For instance, assayed at the optimal pH 8.0, the *K*_*m*_ G6P was significantly lower for anoxic G6PDH as compared with the aerobic control enzyme (Table 1). A similar decrease in *K*_*m*_ G6P in response to anoxia was seen in hepatopancreas of the freshwater crayfish, *Orconectes virilis* (Lant & Storey, 2011) and *K*_*m*_ G6P of hepatopancreas G6PDH also decreased by about one-half when terrestrial snails, *Otala lactea*, entered estivation, a state of aerobic hypometabolism (Ramnanan & Storey, 2006). Hence, an increase in G6P substrate affinity (decreased *K*_*m*_) by hepatopancreas G6PDH seems to accompany a transition into the hypometabolic state in multiple invertebrate species and, therefore, may be important in adjusting enzyme function for an altered role during anaerobiosis, such as a greater involvement in antioxidant defense.

Sensitivity to urea denaturation (lower *I*_50_ value) was also greater for anoxic G6PDH from *L. littorea*. Assayed at pH 6.0, the *K*_*m*_ NADP and the *V*_max_ activity also differed significantly between the aerobic to anoxic states. However, the enzyme from aerobic versus anoxic hepatopancreas did not differ in other parameters including sensitivity to salts and activation energy (Table 1). In addition, the pH curves for both enzymes were generally quite similar (Fig. 1). When the effect of pH on enzyme parameters was considered, both aerobic control and anoxic G6PDH showed a large decrease in affinity (*K*_*m*_ increased 4.2–7.8 fold) when assayed at pH 6 as compared with pH 8. Since physiological pH decreases over time when anaerobiosis is prolonged due to accumulation of acidic end products (Ellington, 1983a; Ellington, 1983b; Pörtner, Grieshaber & Heisler, 1984), this could argue for reduced functionality of G6PDH under anoxic conditions. However, *K*_*m*_ NADPH of anoxic G6PDH was unaltered between pH 8 and 6 whereas low pH elevated this parameter of the control form and also decreased *V*_max_ more strongly than occurred for the anoxic enzyme. On balance, it appears that the anoxic enzyme form has kinetic properties that are better suited for function under the more acidic cellular conditions experienced in the anoxic state and this could aid continued enzyme function in the production of NADPH for use in antioxidant defense.

The stable changes in kinetic parameters that distinguish the aerobic control versus anoxic enzyme forms of *L. littorea* G6PDH suggested that there are physical/structural differences between the two. This was further supported by the very different elution patterns of the two enzyme forms off DEAE-Sephadex (anoxic G6PDH eluting at a much higher salt concentration) (Fig. 2); this indicated that the two enzyme forms possess different surface charges. The differing sensitivity to urea denaturation by the two enzyme forms (Table 1) also supports a structural difference between the enzymes. The greater sensitivity of the anoxic form to urea suggests a more flexible structure than that of the aerobic control enzyme.

The significant stable differences in some of the kinetic and physical properties of hepatopancreas G6PDH between control and anoxic states suggested a potential difference in the covalent modification of the enzyme between the two states. To investigate this we first analyzed the serine phosphorylation state of G6PDH from control and anoxic hepatopancreas and found that control GDH contained a 2.5-fold greater amount of bound phosphate compared with anoxic G6PDH (Fig. 3). Serine phosphorylation of G6PDH has been reported in mammalian systems (Napier et al., 1987; Xu, Osborne & Stanton, 2005; Pan et al., 2009) and reversible phosphorylation of G6PDH has also recently been associated with stress-induced hypometabolism in both vertebrate and invertebrate systems (Ramnanan & Storey, 2006; Dieni & Storey, 2010; Lant & Storey, 2011).

**Figure 6:**
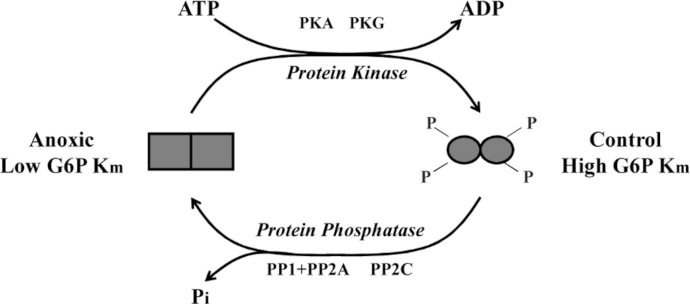
Schematic representation of *L. littorea* hepatopancreas G6PDH control by reversible phosphorylation between normoxic and anoxic conditions. Under normoxic (control) conditions G6PDH is in a more highly phosphorylated form. As this marine snail becomes anoxic, G6PDH becomes dephosphorylated (possibly by PP1, PP2A or PP2C) and this lowers the Km G6P. Upon re-exposure to an oxygen rich environment, G6PDH becomes phosphorylated (possibly by PKA or PKG) and this increases Km G6P.

To further explore *L. littorea* G6PDH phosphorylation, we used *in vitro* incubations that stimulated the activities of endogenous protein kinases (PKA, PKC, PKG) and protein phosphatases (PP1, PP1 + PP2A, PP2B and PP2C) in crude tissue extracts and then evaluated the effects of incubations of *K*_*m*_ G6P. Incubations that stimulated the activities of PKA and PKG caused a significant increase in *K*_*m*_ G6P of anoxic G6PDH to a value similar to that seen for the aerobic control enzyme (Fig. 3A). Conversely, incubations that stimulated PP1, PP1 + PP2A or PP2C caused significant decreases in *K*_*m*_ G6P of aerobic control G6PDH to values similar to those of anoxic G6PDH (Fig. 3B). Figure 6 shows a schematic of this reversible phosphorylation control of G6PDH between normoxia and anoxia. Several previous studies have identified PKA-mediated phosphorylation of G6PDH including the muscle enzyme from a freshwater bivalve mollusc, *Anodonta cygnea* (Kuznetsova & Plesneva, 2001). PKA-mediated phosphorylation also inhibits G6PDH activity from various mammalian sources (Costa Rosa et al., 1995; Zhang et al., 2000; Xu, Osborne & Stanton, 2005). *In vitro* incubations also implicated both PKA and PKG in the phosphorylation of hepatopancreas G6PDH in response to anoxia in crayfish or estivation in a terrestrial snail (Ramnanan & Storey, 2006; Lant & Storey, 2011) although additional studies suggested that PKG was the most likely candidate *in vivo* in the snail. Interestingly, in the marine snail, tissue PKA activity increased during aerobic recovery from anoxia (MacDonald & Storey, 1999), which would fit with a potential role for PKA in phosphorylating anoxic G6PDH when *L. littorea* transitions back to aerobic conditions. PKA and PP1 are often paired in their effects and therefore these might be the primary kinase and phosphatase involved in regulating *L. littorea* hepatopancreas G6PDH *in vivo* in response to aerobic/anoxic conditions.

To confirm that the incubation experiments were indeed responsible for transfering phosphate moieties onto G6PDH, a total kinase incubation was performed in the presence of γ-^32^P-ATP using G6PDH from aerobic control hepatopancreas that was first treated to dephosphorylate the enzyme. Radiolabelled phospho-G6PDH was then immunoprecipitated using a G6PDH antibody and insoluble protein A. These experiments demonstrated that G6PDH incorporated high amounts of ^32^P when incubated under conditions that stimulated protein kinases as compared with incubation under STOP conditions when kinases were inhibited (Fig. 4). These results validate the results from the *in vitro* incubations and demonstrate that periwinkle G6PDH can be phosphorylated, which further confirms that G6PDH is regulated by reversible protein phosphorylation in the hepatopancreas of *L. littorea*.

In summary, the present study shows that G6PDH from *L. littorea* hepatopancreas is a phosphoprotein and that entry into the hypometabolic state of anoxia results in the dephosphorylation of G6PDH. The low phosphate, anoxic form of G6PDH is consistent with a more active enzyme which may favour enhanced carbon flow through the pentose phosphate cycle during anoxia to sustain NADPH production for antioxidant defense. The data provides one of the first demonstrations of coordinated regulation of the pentose phosphate cycle between active and hypometabolic states and implicates specific protein kinases (PKA, PKG) and protein phosphatases (PP1, PP2A, PP2C) as responsible for G6PDH regulation.
